# Techniques for the insertion of the proseal laryngeal mask airway: comparison of the foley airway stylet tool with the introducer tool in a prospective, randomized study

**DOI:** 10.1186/1471-2253-14-105

**Published:** 2014-11-18

**Authors:** Mao-Kai Chen, Hung-Te Hsu, I-Cheng Lu, Chih-Kai Shih, Ya-Chun Shen, Kuang-Yi Tseng, Kuang-I Cheng

**Affiliations:** Department of Anesthesiology, Kaohsiung Medical University Hospital, Kaohsiung, Taiwan; Department of Anesthesiology, Kaohsiung Municipal Ta-Tung Hospital, Kaohsiung, Taiwan; Department of Anesthesiology, Changhua Christian Hospital, Changhua, Taiwan; Department of Anesthesiology, Faculty of Medicine, Kaohsiung Medical University, Kaohsiung, Taiwan

**Keywords:** ProSeal laryngeal mask airway (LMA), Foley Airway Stylet Tool (FAST), Introducer tool (IT), Fiberoptic bronchoscope

## Abstract

**Background:**

Many tools have been developed to facilitate the insertion of the ProSeal laryngeal mask airway (LMA) insertion, which can be impeded by folding of its soft cuff. The aim of this study was to compare the efficiency of ProSeal LMA insertion guided by a soft, direct optical Foley Airway Stylet Tool (FAST) with the standard introducer tool (IT).

**Methods:**

One hundred sixty patients undergoing general anesthesia using the ProSeal LMA as an airway management device were randomly allocated to either FAST-guided or IT-assisted groups. Following ProSeal LMA insertion, the glottic and esophageal openings were identified using a fiberoptic bronchoscope introduced through the airway and the drain tube. The primary outcomes were time taken to insert the ProSeal LMA and the success rate at the first attempt. Secondary end points included ease of insertion, hemodynamic response to insertion, and postoperative adverse events recorded in the recovery room and on the first postoperative morning.

**Results:**

One hundred forty patients were included in the final analysis: 66 in the FAST-guided group and 74 in the IT-assisted group. The success rate of FAST device-guided ProSeal LMA insertion (95.7%) was broadly comparable with IT-assisted insertion (98.7%). However, the time taken to insert the ProSeal LMA was significantly longer when the FAST technique was used (p <0.001). The incidence of correct alignment of the airway tube and the drain tube did not differ significantly between the groups. There were no significant differences in ease of insertion or hemodynamic responses to insertion, except that the incidence of postoperative sore throat was significantly higher in the FAST group on the first postoperative day (22.2% compared with 6.8% in the IT group; p =0.035).

**Conclusion:**

Both FAST-guided and IT-assisted techniques achieved correct ProSeal LMA positioning, but the IT technique was significantly quicker and less likely to cause a sore throat.

**Trial registration:**

ClinicalTrials.gov Identifier: NCT02048657

## Background

The ProSeal laryngeal mask airway (LMA; Orthofix, Maidenhead, UK) is a laryngeal mask with a modified cuff that incorporates a drainage tube to improve the quality of the seal while reducing the risk of pulmonary aspiration and gastric insufflation [1–3]. When the device is used for controlled ventilation, the ProSeal LMA provides a higher oropharyngeal leak pressure than the Classic LMA [4]. However, the ProSeal LMA is reported to be more difficult to insert than the Classic LMA, as its larger, softer cuff is prone to folding. The manufacturer recommends that the ProSeal LMA be inserted using either manipulation with the fingers or a curved metal introducer. Nonetheless, first attempt success rates of ProSeal LMA insertion range from 81% to 87%, which is lower than the Classic LMA [2, 4, 5].

Consequently, a variety of techniques has been developed to facilitate insertion of the ProSeal LMA, including priming the drain tube with a guiding instrument such as a suction catheter [6], a gastric tube [7], a gum elastic Bougie [8], a Flexi-Slip stylet [9] and even a fiberoptic bronchoscope [10, 11]. Most are based on blind catheter or tube insertion, and although a fiberoptic bronchoscope enables the intraoral structures to be viewed, it is too expensive and cumbersome to be used in routine practice. The Foley Airway Stylet Tool (FAST) is a portable, simple and robust battery-powered, flexible fiberoptic endoscope. It has been reported to facilitate tracheal intubation with an intubating LMA [12], and thus we hypothesized that it might also be advantageous for ProSeal LMA insertion. The aim of this study was to compare the efficiency of the FAST optical stylet technique with the standard introducer tool (IT) technique for ProSeal LMA insertion. We determined that the LMA was correctly positioned by checking its alignment with the glottic and esophageal openings using a fiberoptic bronchoscope. We evaluated both techniques in terms of success rates, insertion times, insertion difficulty, hemodynamic response to insertion and the incidence of postoperative sore throat.

## Methods

**Figure 1:**
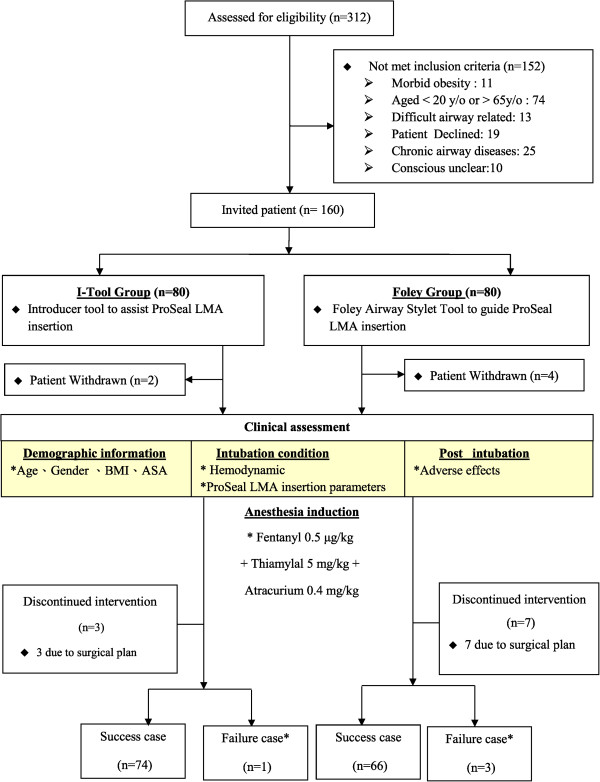
**Consolidated Standards of Reporting Trials (CONSORT) diagram for the study.**

The study was approved by our local ethics committee (Kaohsiung Medical University Hospital, Kaohsiung City, Taiwan, KMHK-IRB 96023) and informed consent was obtained from all patients and got ClinicalTrials.gov Identifier (NCT02048657). We enrolled 160 adults (American Society of Anesthesiologists (ASA) physical status I-II, aged between 20 and 65 years) scheduled for elective surgery under general anesthesia using the ProSeal LMA for airway management. Exclusion criteria included an anticipated difficult airway, morbid obesity, inadequate fasting, and pre-existing sore throat or hoarseness. Patients were allocated randomly into one of two groups using a computer-generated random number table. The FAST was used to guide ProSeal LMA insertion in 80 patients (FAST group) and the ProSeal LMA was inserted with a standard IT in the remaining 80 patients (IT group) by one of two experienced anesthesiologists (Figure 1). None of the patients was aware of the insertion method used.

No premedication was administered. In the operating room, heart rate, blood pressure and arterial oxygen saturation were recorded at baseline and then every 5 min thereafter. Each patient was anesthetized in the supine position with the head resting on a 7-cm high pillow. The fluid balance deficit resulting from the overnight fast was addressed by administering lactated Ringer’s solution at one-third of the patient’s estimated circulating volume preoperatively. The size of the ProSeal LMA was selected according to the patient’s weight: size 3 for those ≤50 kg, size 4 for those >50 kg). The cuff was fully deflated and the back surface lubricated. Fentanyl 0.5 μg/kg and thiamylal 5 mg/kg were administered as induction agents, and atracurium 0.4 mg/kg given to facilitate ProSeal insertion. Following 3 minutes of mask ventilation with 60% oxygen, the ProSeal LMA was inserted using either the FAST or IT technique. Thereafter, sevoflurane was administered at an end-tidal concentration of 1.5% to 2% and end tidal CO_2_ (ETCO_2_) concentration maintained within the range 35 mmHg to 40 mmHg.

**Figure 2:**
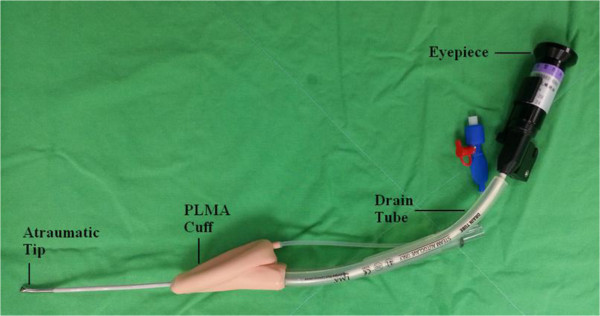
**Settings of the Foley Airway Stylet Tool® (FAST) in a ProSeal LMA.** The atraumatic tip of the stylet can be seen protruding from the distal end of the drainage tube; the flexible portion of the stylet extends 5–6 cm beyond the cuff.

The FAST device (Foley Airway Stylet Tool®, Clarus Medical, Minneapolis, MN, USA) was lightly lubricated and passed down the drainage tube until the tip of the FAST had completely emerged from its distal end (Figure 2). The FAST technique was performed thus: (1) an assistant opened the patient’s mouth; (2) the anesthesiologist held the distal portion of the stylet in the nondominant hand while holding the eyepiece of the FAST device in the dominant hand; (3) the atraumatic tip was inserted into the esophageal opening under direct vision of the laryngo-pharyngeal tissues through the patient’s mouth; (4) the anesthesiologist introduced the lubricated ProSeal LMA into the pharynx along the FAST stylet; and finally (5) the FAST device was removed while the ProSeal LMA was held in place.

In the IT group, the ProSeal LMA was inserted using the metal introducer according to the manufacturer’s instructions: (1) the patient’s mouth was opened; (2) the lubricated ProSeal LMA was inserted into the patient’s mouth using the introducer; (3) the ProSeal LMA was advanced using a one-handed technique until resistance was encountered; and (4) the introducer was removed, leaving the ProSeal in position.

Once the ProSeal LMA had been positioned in the pharynx, the cuff was inflated with air according to a pressure of 60 cmH_2_O. Two attempts were allowed before insertion was considered to have failed. Failed insertion was defined by any of the following criteria: (1) failure to advance the ProSeal LMA into the pharynx; (2) ProSeal LMA malposition (air leaks despite cuff inflation); or (3) ineffective ventilation (maximum expired tidal volume <6 ml/kg).

Insertion time was defined as time elapsed from opening the mouth until the ProSeal LMA was connected to the anesthetic breathing circuit. Heart rate and blood pressure were measured before and after ProSeal LMA insertion. Ease of insertion was graded as smooth, mildly resistant or requiring a second attempt. The quality of the fit of the ProSeal LMA in the glottis was evaluated using a fiberoptic bronchoscope (Olympus LF-2, Shinjuku, Tokyo, Japan) at the laryngeal aperture of the ProSeal LMA through the airway tube. The glottic view was scored using a five-point scale (Table 1) based on previous studies [2, 13, 14]. The alignment of the tip of the drain tube with the esophageal opening was evaluated using a fiberoptic bronchoscope inserted in the drain tube. The view of the esophagus was scored using a three-point scale (Table 1). Postoperative sore throat was recorded in the postoperative care unit and on the following morning. All parameters were recorded and data analyzed by an investigator blinded to the insertion technique.

**Table 1:** **Grading of the views of the laryngeal field from the distal aperture of the airway tube and the esophagus from the drain tube using fiberoptic bronchoscopy**

Viewing	Grading	Description
Laryngeal field	**1**	**Vocal cords not seen and device functions inadequately**
	**2**	**Vocal cords not seen but device functions adequately**
	**3**	**Vocal cords and anterior epiglottis**
	**4**	**Vocal cords and posterior epiglottis**
	**5**	**Only vocal cords visible**
Esophagus	**A**	**Sealed orifice of esophagus**
	**B**	**Crescent shape opening of esophagus**
	**C**	**Full opening of esophagus**

Study design was informed by the findings of a previous report of Bougie-guided LMA insertion time [8], and sample size estimated on the basis of one control per experimental subject, a difference in mean insertion time of 10 seconds between groups, normally distributed data and a standard deviation of 17 seconds. At least 62 subjects were needed in each group based on a type I error 0.05 and a power of 0.9. We allocated 80 patients to each group to take into account the possibility of surgical problems and failed ProSeal LMA insertion requiring patients to be excluded from analysis of total insertion time to find out reasons. We compared patient characteristics, insertion time, insertion attempts, adverse effects and complications between the groups using the two-sample *t*-test (for numerical variables) and the Chi-square test (for categorical variables). Statistical significance was set at a p value <0.05.

## Results

From June 2008 to Dec 2009, fourteen patients in the FAST group were excluded from final analysis (seven owing to changes in the surgical plan, four withdraw consent, and three owing to failed insertion). Of the three failed insertions, two in the FAST group were as a consequence of the cuff folding in the oropharynx and one owing to an unexpectedly blurred view through the FAST device during insertion. Six patients in the IT group were excluded from final analysis (three owing to a change in surgical plan, two withdraw consent, and one owing to a failed insertion caused by folding back of the ProSeal LMA and air leak). Therefore, 69 patients in the FAST group and 75 patients in IT the group were included in the primary analysis (Figure 1). Sixty-six patients in the FAST group and 74 patients in the IT group were included in secondary analysis for ProSeal LMA insertion time spent and adverse events. There were no differences between the groups in terms of age, sex, height, weight, body mass index, ASA physical status or size of ProSeal LMA used (Table 2).

**Table 2:** **Patient characteristics, ASA classification, LMA size, and duration and specialty of surgery**

	FAST group (n = 69)	IT group (n = 75)	P values
Male/female	16/53	28/47	0.066
Age (yr)^#^	41 (20-65)	43 (22-65)	0.452
Height (cm)	159.5 (8.4)	160.7 (8.0)	0.360
Weight (kg)	61.4 (13.3)	62.6 (11.2)	0.573
BMI	24.0 (4.0)	24.2 (3.5)	0.863
ASA I/ II	35/34	31/44	0.259
LMA size NO:3/NO:4	26/43	23/52	0.375
Duration of surgery (min)	117 (53)	120 (51)	0.717
Type of surgery			
Otorhinolaryngology	3	1	0.182
Gynecology	29	26	
Orthopedic	21	29	
General	16	15	
Urology	0	4	

^#^Median [95% confidence interval]; BMI: body mass index; ASA, American Society of Anesthesiologists; SD: standard deviation.

All data are shown as mean (SD) unless otherwise stated.

The hemodynamic responses to ProSeal LMA insertion were broadly comparable between the groups: there were no significance differences in heart rate or mean arterial pressure (Table 3).

**Table 3:** **Hemodynamic responses to ProSeal laryngeal mask airway insertion**

	FAST group	-Tool group	P value
Number of patients	66	74	
Heart rate			
Before	80.8 (14.1)	79.1 (15.6)	0.97
After	77.8 (12.8)	75.5 (14.7)	0.33
Mean arterial pressure			
Before	99.2 (13.9)	99.1 (15.4)	0.82
After	101.1 (15.4)	98.3 (17.8)	0.27

The success rate of ProSeal LMA insertion was 95.7% in the FAST group and 98.7% in the IT group (p =0.26), and insertion took significantly longer when the FAST technique was used (p <0.001; Table 4). The ability to visualize the glottic structures and esophageal opening was also not significantly different between the groups (Table 4). The incidence of sore throat was higher in the FAST group than the IT group on the next postoperative day (p =0.035).

**Table 4:** **ProSeal LMA insertion parameters and incidence of postoperative sore throat**

	FAST group	IT group	P value
Insertion time	17.4 ± 6.1	12.6 ± 4.7	<0.001^***^
Insertion condition			
Smooth	62 (89.9%)	67 (89.4%)	
Mild resistance	2 (2.9%)	1 (1.3%)	0.413
Two attempts	2 (2.9%)	6 (8.0%)	
Failed insertion^#^	3 (4.3%)	1 (1.3%)	
Viewing glottic field			
1	0 (0%)	0 (0%)	0.201
2	4 (6.1%)	2 (2.7%)	
3	42 (63.6%)	57 (77.0%)	
4	20 (30.3%)	15 (20.3%)	
5	0 (0%)	0 (0%)	
Viewing esophagus			
A	59 (89.4%)	69 (93.2%)	0.435
B	6 (9.1%)	3 (4.1%)	
C	1 (1.5%)	2 (2.7%)	
Sore throat in PACU			
None	44 (66.7%)	56 (75.7%)	0.377
Mild	20 (30.1.1%)	18 (24.3%)	
Moderate	1 (1.5%)	0 (0%)	
Severe	1 (1.5%)	0 (0%)	
Sore throat on the first			
Postoperative morning			
None	52 (78.8%)	69 (93.2%)	0.035^*^
Mild	11 (16.7%)	3 (4.1%)	
Moderate	3 (4.5%)	2 (2.7%)	
Severe	0 (0%)	0 (0%)	

PACU, post-anesthesia care unit; #: Due to failed insertion, ProSeal LMA post-insertion assessments were excluded. *: *P*< 0.05; ***: *P*< 0.001.

## Discussion

We found that the ProSeal LMA can be properly positioned using either the FAST-guided technique or IT techniques; fiberoptic bronchoscopy showed that the mask aligned correctly with the glottis and esophagus in the majority of cases. The success rates of ProSeal LMA insertion by each technique were broadly comparable, and both exceeded 95%. Nevertheless, the FAST technique took significantly longer and the incidence of sore throat on the second postoperative day was significantly higher than the traditional IT technique. We can therefore draw the conclusion that the direct vision FAST device does not appear to have any advantages over the traditional introducer for ProSeal LMA insertion.

A popular method of facilitating ProSeal LMA insertion is to use a gum-elastic Bougie in the drainage tube together with a laryngoscope, which allows the tip to be correctly positioned in the esophagus. This technique is recommended as a second-line backup means of directly visualizing the upper airway [8]. The Bougie provides sufficient rigidity to guide the cuff directly into the pharynx without folding [8, 15, 16]. However, without laryngoscopic assistance, blind insertion is reported to have caused pharyngeal wall perforation [17].

The FAST device is a lighted fiberoptic malleable stylet that functions as an “optical Bougie” and has several advantages that make it suitable for airway management: it is flexible and portable, has an atraumatic tip, and can be used easily by a single operator. These advantages ensure that the drain tube aligns with the esophageal opening during ProSeal LMA insertion. The device has been reported to aid endotracheal intubation through the intubating LMA with a success rate of 90% (27 out of 30 patients) at the first attempt and 96.7% after subsequent attempts [12]. For ProSeal LMA insertion, the FAST device provides direct vision to guide passage of the airway and prevent cuff folding or impaction during manipulation. Our findings did not support the hypothesis that the FAST device was superior to the traditional introducer-assisted technique, but it may still be useful for some instances of ProSeal LMA insertion.

We did not use a size 5 ProSeal LMA in patients weighing more than 80 kg as some patients’ passive mouth opening was not sufficient to accommodate a mask of that size. We prefer to use a smaller ProSeal LMA in such patients, which in our experience is easier to insert. Six patients in FAST group and eight patients in the IT group weighed >80 kg, respectively, and in all cases insertion was successful at the first attempt. Brimacombe and Keller have reported the successful use of a size 4 ProSeal LMA in patients within a height range of 150–193 cm and weight rang of 40–115 kg [2]. A variety of means of selecting LMA size on the basis of sex [18, 19], height [20], or an algorithm [21, 22] have been described. In apneic anesthetized adults weighing <100 kg, the optimal LMA size is reported to be size 5 in 63% and size 4 in 36% [20]. Determining the optimal size LMA for an individual adult is complex, and the best strategies for selecting either size 4 or 5 LMA in adults should be investigated in more detail.

There are several possible explanations for the folding of the cuff in the oropharynx that we observed in two patients in the FAST group despite guidance with the flexible stylet. First, the need for the operator to use two hands may complicate insertion; the dominant hand holds the eyepiece firmly but overly rapid withdrawal of the stylet by the nondominant hand might displace the LMA from its correct position. Second, an inadequate length of insertion of the stylet might easily dislodge the tip from the esophagus. Third, secretions may obscure the visual field and impede advancement of the device. Fourth, the unidirectional tip could obscure the visual field causing the tip to impact on the pharyngeal wall. Neither the FAST device nor the introducer tool can completely eliminate folding of the cuff during ProSeal LMA insertion.

Sore throat may be caused by friction between the ProSeal LMA cuff and oropharyngeal tissues during placement and removal, high cuff inflation pressure, forceful LMA advancement, or advancement of the FAST stylet tip. When injury occurs, it is usually manifest as a minor complaint such as dry mouth or sore throat [23]. In this study, the cuff of the ProSeal LMA was inflated to 60 cmH_2_O. The incidence of sore throat on the second postoperative day was 21.2% in the FAST group and 6.8% in the IT group, which is similar to that reported by other investigators [24, 25]. Although the exact mechanisms of ProSeal LMA-induced sore throat have yet to be identified, the finding that sore throat is also common after upper gastrointestinal endoscopy suggests that instrumentation of the airway is more likely to be responsible than pressure from the cuff once it is *in situ* [26]. This may also help explain the increased incidence of sore throat after FAST-guided ProSeal LMA insertion that we observed.

Our study had some limitations. First, all ProSeal LMA insertions were undertaken by experts (>1,000 cases) who clearly could not be blinded to the insertion technique, so our findings may not apply to inexperienced users. Second, the use of muscle relaxants meant that there was minimal resistance in the oropharynx and larynx during ProSeal LMA insertion, and only mild airway trauma was encountered. This may not be the case in spontaneously breathing patients. Third, patients with anticipated difficult airways were excluded, so our findings may only apply to patients with normal airways. Fourth, we found that the unidirectional tip of the FAST device limited our ability to direct the LMA during insertion, likely causing insertion failure in three cases. The incidence of failed insertion is likely to be higher in patients with anticipated difficult intubation.

## Conclusion

Both the FAST device and IT technique can achieve accurate ProSeal LMA positioning with similar success rates. However, for inexperienced users the FAST device may not hold any advantages over the standard introducer-guided technique. Introducer-tool assisted ProSeal LMA insertion is quicker and is associated with a lower incidence of sore throat.

## Abbreviations

LMA: Laryngeal mask airway
FAST: Foley airway stylet tool
IT: Introducer tool
ETCO_2_: End tidal CO_2_.
